# Prevention of persistent pain with lidocaine infusions in breast cancer surgery (PLAN): study protocol for a multicenter randomized controlled trial

**DOI:** 10.1186/s13063-024-08151-4

**Published:** 2024-05-22

**Authors:** James S. Khan, Ian Gilron, P. J. Devereaux, Hance Clarke, Nour Ayach, George Tomlinson, May Lynn Quan, Karim S. Ladha, Stephen Choi, Allana Munro, Richard Brull, David W. Lim, Sinziana Avramescu, Philippe Richebé, Nicole Hodgson, James Paul, Daniel I. McIsaac, Simone Derzi, Geoff L. Zbitnew, Alexandra M. Easson, Naveed T. Siddiqui, Sarah J. Miles, Keyvan Karkouti, PLAN Trial Investigators, Elena Parvez, Nicole J. Look Hong, Frances C. Wright, Amanda Roberts, Jaime Escallon, Gary Ko, Alexander Huang, Fabricio B. Zasso, Wey L. Leong, Andrea M. Covelli, Howard Meng, Ana Sjaus, Tina Kerelska, Vishal Uppal, Yehoshua Gleicher, Anne O’Neill, Li Wang, Daniel Sellers, Maria B. C. Chuquer, Geoffrey S. Hawboldt, Stefan O. P. Hofer, Harsha Shanthanna, Lucy K. Helyer, Bilal M. Ansari, Salima S. J. Ladak, Inna Oyberman, Erin Cordeiro, Carlos A. Ibarra Moreno, Elad Dana, Jason W. Busse, D Norman Buckley, Siba Haykal, Stuart A. McCluskey, Dolores McKeen, Julian Wiegelmann, Geoffrey Warden, Kathryn A. Sparrow, Mandeep Singh, Rachael Bosma, David Flamer, Richard L. Mah, Derek Diliane, Antoine Bouchard-Fortier, Alison Laws, Ashley Drohan

**Affiliations:** 1 Department of Anesthesiology & Pain Medicine, Mount Sinai Hospital, University of Toronto, Toronto, ON Canada; 2 Departments of Anesthesiology & Perioperative Medicine, and Biomedical & Molecular Sciences, Centre for Neuroscience Studies, and School of Policy Studies, Queen’s University and Kingston Health Sciences Centre, Kingston, ON Canada; 3 Population Health Research Institute, McMaster University, Hamilton Health Sciences Corporation, Hamilton, ON Canada; 4 Department of Anesthesiology and Pain Medicine, University of Toronto, Toronto, ON Canada; 5 Transitional Pain Service, Toronto General Hospital, Toronto, ON Canada; 6 Department of Anesthesiology & Pain Medicine, University of Toronto, Toronto, ON Canada; 7 Department of Medicine, University Health Network and Mount Sinai Hospital, Toronto, ON Canada; 8 University of Toronto, Toronto, ON Canada; 9 Department of Surgery/Oncology, University of Calgary, Calgary, AB Canada; 10 Department of Anesthesia at St. Michael’s Hospital, Toronto, ON Canada; 11 Department of Anesthesiology and Pain Medicine, Sunnybrook Health Sciences Centre, University of Toronto, Toronto, ON Canada; 12 Department of Anesthesia, Pain Management, and Perioperative Medicine, Dalhousie University, Halifax, NS Canada; 13 Department of Anesthesiology and Pain Medicine, Women’s College Hospital, University of Toronto, Toronto, ON Canada; 14 Women’s College Research Institute & Department Surgery, Women’s College Hospital, Toronto, ON Canada; 15 Department of Anesthesiology and Pain Medicine, Humber River Hospital, University of Toronto, Toronto, ON Canada; 16 Department of Anesthesiology and Pain Medicine, Maisonneuve-Rosemont Hospital, CIUSSS de L’Est de L’Ile de Montreal (CEMTL), University of Montreal, Montreal, QC Canada; 17 Department of Surgery, McMaster University, Hamilton, ON Canada; 18 Department of Anesthesia, McMaster University, Hamilton, ON Canada; 19 Departments of Anesthesiology & Pain Medicine and School of Epidemiology & Public Health, The Ottawa Hospital, University of Ottawa, Ottawa, ON Canada; 20 Department of Anesthesiology & Pain Medicine, University of Alberta, Edmonton, AB Canada; 21 Department of Anesthesiology, Memorial University, St. John’s, NF Canada; 22 Department of Surgery and Institute of Health, Policy, Management and Evaluation (HPME), Mount Sinai Hospital and Princess Margaret Cancer Centre, University of Toronto, Toronto, ON Canada; 23 Department of Anesthesia and Pain Medicine, University of Toronto, Toronto, ON Canada; 24 Department of Anesthesia and Pain Management, University Health Network, Sinai Health System, and Women’s College Hospital, Toronto, ON Canada

**Keywords:** Chronic pain, Chronic postsurgical pain, Perioperative care, Breast cancer surgery, Lidocaine infusion, Post-mastectomy pain syndrome

## Abstract

**Background:**

Persistent pain is a common yet debilitating complication after breast cancer surgery. Given the pervasive effects of this pain disorder on the patient and healthcare system, post-mastectomy pain syndrome (PMPS) is becoming a larger population health problem, especially as the prognosis and survivorship of breast cancer increases. Interventions that prevent persistent pain after breast surgery are needed to improve the quality of life of breast cancer survivors. An intraoperative intravenous lidocaine infusion has emerged as a potential intervention to decrease the incidence of PMPS. We aim to determine the definitive effects of this intervention in patients undergoing breast cancer surgery.

**Methods:**

PLAN will be a multicenter, parallel-group, blinded, 1:1 randomized, placebo-controlled trial of 1,602 patients undergoing breast cancer surgery. Adult patients scheduled for a lumpectomy or mastectomy will be randomized to receive an intravenous 2% lidocaine bolus of 1.5 mg/kg with induction of anesthesia, followed by a 2.0 mg/kg/h infusion until the end of surgery, or placebo solution (normal saline) at the same volume. The primary outcome will be the incidence of persistent pain at 3 months. Secondary outcomes include the incidence of pain and opioid consumption at 1 h, 1–3 days, and 12 months after surgery, as well as emotional, physical, and functional parameters, and cost-effectiveness.

**Discussion:**

This trial aims to provide definitive evidence on an intervention that could potentially prevent persistent pain after breast cancer surgery. If this trial is successful, lidocaine infusion would be integrated as standard of care in breast cancer management. This inexpensive, widely available, and easily administered intervention has the potential to reduce pain and suffering in an already afflicted patient population, decrease the substantial costs of chronic pain management, potentially decrease opioid use, and improve the quality of life in patients.

**Trial registration:**

This trial has been registered on clinicaltrials.gov (NCT04874038, Dr. James Khan. Date of registration: May 5, 2021).

**Supplementary Information:**

The online version contains supplementary material available at 10.1186/s13063-024-08151-4.

## Background

### Persistent pain after breast cancer surgery

Breast cancer is the most commonly diagnosed cancer globally (2.3 million cases annually; ~ 12% of cancer diagnoses) [1] and the second most common type of cancer diagnosed in Canada (28,000 cases annually), despite it almost exclusively affecting females [2]. Fortunately, advances in cancer screening and management have substantially improved its prognosis, leading to a 85-90% 5-year survival rate in developed countries [1]. Increased survivorship has led, however, to the identification of important and problematic long-term complications from breast cancer management, specifically the devastating effects of persistent pain.

Breast cancer surgery is a critical component to breast cancer treatment. A common, yet vastly under-recognized complication after breast cancer surgery, including mastectomy and segmental mastectomy (lumpectomy), is persistent pain, often referred to as post-mastectomy pain syndrome (PMPS). PMPS is pain around the surgical incision (axilla, medial arm, shoulder, or chest wall on the side of surgery) that does not resolve, or worsens, ≥ 3 months after surgery (time needed to heal from a surgical incision) [3–5]. Unfortunately, breast cancer surgery has one of the highest rates of persistent pain among all surgical sub-types [6, 7]. In a recent systematic review of patients undergoing breast cancer surgery (146 studies, *n* = 137,675), the prevalence of persistent pain after breast cancer surgery was 35% (95% confidence interval [CI] 32 to 39%) [8]. Further, 20% of patients with persistent postsurgical pain report that their pain is moderate to severe in intensity (numeric rating scale [NRS] ≥ 4) [8–10]. Persistent pain after breast cancer surgery is chronic—over 50% of those afflicted will continue to suffer from it 7–12 years after surgery [11]. These are staggering statistics given that > 22,000 Canadians undergo breast cancer surgery each year [2] and younger patients (< 40 years) are at greater risk of this complication [12]. Persistent pain has a tremendous impact on quality of life as it causes emotional distress, mood disturbance, impaired sleep, and reduced physical functioning [13–16]. These deleterious effects of PMPS are experienced at a societal level through direct (healthcare costs) and indirect (loss of productivity) expenses, totaling $1 billion USD a year in the USA alone [17].

Although the pathophysiology of PMPS is not fully understood and likely multifactorial in nature, the inciting event may be related to neuronal injuries during surgical resection of cancerous tissue [4, 12, 18–21]. When peripheral nerves are injured, particularly nerves involved in pain transmission (A-δ and C-fibers), they respond with a barrage of neurochemical discharges [22, 23]. Given the rich innervation of the breast tissue and chest wall with peripheral nerves, trauma to these nerves during the perioperative care can initiate peripheral neuroplastic changes that ultimately lead to upregulation of pain receptors and mediators along the central nervous system (central sensitization), which is a requirement in the development of persistent and refractory pain [24].

### Intravenous lidocaine infusions

Intravenous lidocaine has been used in the treatment for acute pain, cancer pain, neuropathic pain, complex regional pain syndrome, and diabetic neuropathy [25–30]. Lidocaine infusions provide long-term analgesic benefits months after infusion cessation, suggesting a possible reversal effect within the peripheral and central nervous system [31]. While systemic lidocaine can *reverse* neuroplastic pain changes, whether it can be used to *prevent* persistent pain before it occurs is unknown. There is growing evidence to support this hypothesis [32–36]. In various animal models, lidocaine prevents the generation of spontaneous pain signals and the development of peripheral and central pain sensitization [37–39]. A recent meta-analysis of perioperative lidocaine infusion in noncardiac surgeries (6 including 4 in breast cancer surgery, *n* = 420) found that lidocaine significantly reduced the prevalence of chronic postsurgical pain (≥ 3 months after surgery; 101 events; odds ratio [OR] 0.29; 95% CI 0.18 − 0.48; *I*^2^ = 0%) [40]. In addition, IV lidocaine infusion has been explored widely for improved postoperative pain and accelerated recovery after gastrointestinal surgeries [31, 41–46].

Prior RCTs have examined the effect of an intraoperative lidocaine infusion on persistent pain after breast cancer surgery. In a systematic review (97 patients), intraoperative lidocaine compared to placebo significantly reduced persistent pain 3 to 6 months after breast cancer surgery (23 events; relative risk [RR] 0.33; 95% CI 0.14−0.78) [47]. Results from the 3 RCTs published since this review are in line with these results [36, 48, 49]. Kim et al. (78 patients) showed that intraoperative lidocaine decreased pain scores at 3 months after surgery (McGill pain questionnaire 8.9 vs. 12.7, *p* = 0.046) [34]. Kendall et al. (150 patients) found that intraoperative lidocaine decreased the proportion of patients who reported pain attributable to surgery at 6 months after surgery (13% vs. 29%, *p* = 0.04); however, they did not find a difference at 3 months [49]. Similarly, we carried out a pilot RCT (100 patients) demonstrating that lidocaine reduced the development of persistent pain at 3 months by 32% (43% vs. 63%; RR 0.68, 95% CI 0.47 to 1.0; *p* = 0.049) [36]. The small sample sizes in the above RCTs are at risk of chance findings, overestimation of treatment effects, fragility, and limitations in exploring important secondary outcomes such as safety, persistent opioid use, function, and quality of life [40, 50]. Further, these trials were typically conducted at single institutions, limiting generalizability. Lidocaine infusions are a promising intervention to reduce persistent pain after breast cancer surgery; however, before widespread use can be recommended, a large, multicenter, RCT is needed to definitively determine its efficacy.

**Fig. 1:**
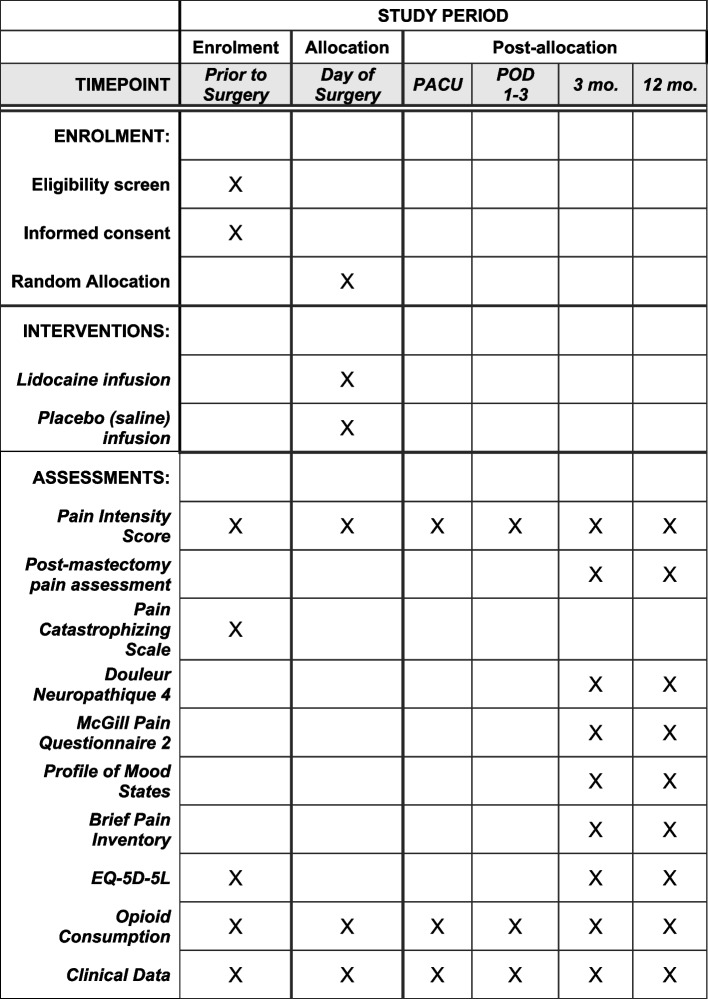
Trial schedule of enrolment, interventions, and assessments

Following our recently published pilot trial demonstrating feasibility, we propose to undertake a Canadian-led, multicenter, international, blinded, randomized, placebo-controlled trial to determine the definitive effects of an intraoperative lidocaine infusion in 1602 patients undergoing breast cancer surgery. This trial is the Prevention of persistent pain with LidocAine iNfusions in breast cancer surgery (PLAN) trial. We used the SPIRIT checklist when writing our report (See Fig. 1) [51].

## Methods

### Trial design

PLAN will be a multicenter, parallel-group, blinded, 1:1 randomized, placebo-controlled, superiority trial of 1602 patients undergoing breast cancer surgery.

### Trial objectives

#### Primary objective

The primary objective of this trial is to determine the effect of an intraoperative intravenous (IV) lidocaine infusion on reducing the development of persistent pain 3 months after breast cancer surgery.

#### Secondary objectives

Secondary objectives of this trial are to measure the effect of IV lidocaine, compared to placebo, on:

- Pain intensities (NRS 0-10) at rest and movement on postoperative days (PODs) 1–3;
- Oral morphine-equivalent opioid consumption on POD 1–3, 3 months, and 1 year after surgery;
- Persistent pain at 1 year after surgery;
- Moderate-to-severe persistent pain (NRS ≥ 4 at rest) at 3 months and 1 year after surgery;
- Persistent neuropathic pain (defined by Douleur Neuropathique 4 [DN4] symptoms assessment) at 3 and 12 months after surgery;
- Sensory and affective qualities of pain (Short Form McGill Pain Questionnaire 2) at 3 months and 1 year after surgery;
- Emotional functioning (Profile of Mood States [POMS]) at 3 months and 1 year after surgery;
- Physical functioning (Brief Pain Inventory [BPI]) at 3 months and 1 year after surgery;
- Quality of life (EQ-5D-5L) at 3 months and 1 year after surgery;
- Cancer recurrence at 12 months;
- Adverse events; adverse drug related events within 24 h of infusion and serious adverse events throughout the trial conduct;
- Cost-effectiveness.

## Participants, interventions, and outcomes

### Study setting

Patients are currently recruited from academic and community hospitals across Canada using a convenience sample of those undergoing breast cancer surgery. Additional international centers are being evaluated to join as recruitment centers to improve generalizability of results.

### Eligibility criteria

#### Inclusion criteria

1. Age ≥ 18 years old; and
2. Undergoing a unilateral or bilateral lumpectomy or mastectomy, inclusive of all pathologies, including prophylactic surgery (e.g., family history or BRCA gene mutation).

#### Exclusion criteria

1. Previous breast surgery within 6 months of index surgery;
2. Undergoing any autologous flap procedure during index surgery;
3. Presence of known chronic pain disorder involving surgical site or ipsilateral chest wall, shoulder, or arm during the 3 months prior to index surgery;
4. Documented hypersensitivity or allergy to lidocaine;
5. Surgery not planned to be performed under general anesthesia and/or planned use of regional or neuraxial anesthetic techniques before surgery (i.e., epidural, paravertebral, serratus plane block, pectoralis or modified pectoralis block);
6. History of ventricular tachycardia, ventricular fibrillation, or atrioventricular block without a pacemaker;
7. Known cirrhotic liver disease;
8. Pregnant; or
9. Unlikely to comply with follow-up (e.g., no fixed address, language difficulties that would impede valid completion of questionnaires, plans to move out of town).

### Randomization

A statistician not involved in the study analysis will generate an online encrypted randomization list. Randomization will use permuted blocks of varying size and will be stratified by center and breast reconstruction (i.e., immediate reconstruction surgery with implants at the time of index surgery). Patients will be randomized in a 1:1 ratio between lidocaine and placebo. Research assistants (RAs) will randomize patients through an online 24-h, interactive, web-based randomization system. Randomization will occur on the day of surgery to minimize the risk of surgery being postponed or cancelled after randomization. For cases performed first thing in the morning, if needed patients could be randomized the night before.

### Blinding

In addition to randomization, we will blind patients, healthcare providers, research assistants, data collectors, investigators, and outcome adjudicators to treatment allocation using blinded study medications. RAs randomizing a patient will receive a unique ID (patient study number) and an encrypted code corresponding to a study medication on the master randomization list. A designated pharmacist at each study center will have access to the master randomization list with encrypted codes and will prepare either a 50-mL syringe of 2% lidocaine or 0.9% normal saline solution. All syringes will be blinded and will indicate study information, the encrypted code and that the enclosed solution is a 2% (or 20 mg/mL) concentration (ensuring equivalent volumes are administered in both treatment groups). The study pharmacist at each participating site will be instructed on the importance of not sharing the blinded allocation data with anyone and will sign a form indicating that they will keep this information confidential.

### Description of study interventions

A study team member (RA or pharmacist) will provide anesthesiologists with study medications for administration. Patients in the intervention group will receive an IV lidocaine infusion using a dosage regimen of 1.5 mg/kg bolus of a 2% lidocaine solution with induction of general anesthesia followed by a 2.0 mg/kg/h infusion until the end of surgery (and up to 30 min in the recovery room). Patients in the control group will receive a placebo bolus and infusion with normal saline (0.9% sodium chloride solution) at the same rate. Study medications will be prepared in blinded 50-mL syringes and labelled as per Health Canada requirements. Total body weight is used for the bolus and infusion dosage calculation. If a patient’s total body weight is over 100 kg, a weight of 100 kg can be used in the calculation, at the discretion of the attending anesthesiologist.

There are no modifications to study infusion regimen allowed. However, if the clinician team suspects a case of local anesthetic toxicity, then the care team can stop the infusion at their discretion and treat the patient according to standard clinical practice guidelines.

Given that study medications are administered by the attending anesthesiologist, compliance with study interventions will be high. In our pilot trial, we had a 94% compliance rate. To ensure compliance with study drug administration in this definitive trial, we have educated the anesthesia and surgical groups at each site through department and grand rounds presentations, provided posters within the operating room, sent reminder emails the day prior for the surgeon and anesthesiologists caring for the included patients, and have our research team present for the start of the surgical case to remind and ensure study medications are being administered.

### Concomitant care

Study patients will be restricted on any additional IV or regional local anesthetic to control the risk of overdose and confounding treatment effects and intraoperative wound infiltration will be limited to 50 mg of a long-acting local anesthetic (i.e., bupivacaine or ropivacaine). Otherwise, intraoperative and postoperative pain management plans will be left to the discretion of the attending clinicians. Our large sample size should balance variations in practices across treatment groups.

### Outcomes

#### Primary outcome

Our primary outcome, persistent pain at 3 months, is informed by the definition for chronic postsurgical pain established by the International Association for the Study of Pain (IASP) [5]. A patient will meet the primary outcome if (1) the patient reports non-zero pain (NRS pain score > 0 at rest) within the last 7 days, (2) located around the surgical incision (i.e., axilla, medial arm, shoulder, or chest wall on the side of surgery), (3) at 3 months after surgery (IASP’s time required for normal healing from injury), (4) with no other identifiable cause of pain (e.g., no pre-existing pain condition, infection, or malignancy).

The NRS scale used in criterion 1 is an 11-point scale where 0 is “no pain” and 10 is “worst possible pain”. Criteria 4 is met by excluding patients with known preoperative pain in the area of their surgery (exclusion criteria) and those with any current or previous (within 1 month of assessment) surgical site infections or known cancer recurrence. If any infections or recurrence is suspected during follow-up, RAs will review with their respective Site Principal Investigator (PI) and send the patient’s medical records to the Anesthesia Clinical Trials Unit (ACTU). If needed, adjudication by two independent investigators blinded to the patient’s treatment allocation will be performed.

#### Secondary outcomes

Study personnel will follow patients (1 h into recovery room stay, PODs 1–3) and ensure the following secondary outcomes are collected, including from the patient’s medical record and pain diary: oral morphine-equivalent opioid consumption; NRS pain scores at rest and movement (taken once in recovery and twice a day [morning and evening] on PODs 1–3); and time to recovery room and hospital discharge. Pain intensity ratings on movement will be assessed by asking the patient to abduct their arm 90° on the ipsilateral side of surgery.

One of the secondary outcomes includes persistent pain at 12 months, which will be assessed using the same criteria as the primary outcome but at 12 months. Moderate-to-severe persistent pain is a binary outcome measured at 3 and 12 months defined as persistent pain with an NRS pain score of ≥ 4 at rest 24 hours before evaluation. Similarly, persistent neuropathic pain is a binary outcome defined as persistent pain at 3 or 12 months that involves neuropathic pain as indicated by the DN4 interview, a validated instrument to detect neuropathic pain [52]. The original DN4 questionnaire required a patient examination but the DN4-interview only requires self-reported data. Patients who meet the criteria for persistent pain at 3 or 12 months after surgery report on the sensory and affective qualities of their pain using the SF-MPQ-2, a reliable tool to evaluate pain in those with chronic pain. It has been validated in a breast cancer population and has been used in several analgesic trials after breast cancer surgery [53–56].

Additionally, all patients will be asked about their emotional and physical functioning, and their quality of life at the 3- and 12-month follow-up. Emotional functioning will be evaluated using the POMS, which is a reliable instrument, validated in a breast cancer population to evaluate aspects of psychological distress (e.g., depression, anxiety, anger) [57]. Physical functioning will be measured by the interference scale of the BPI, which provides a reliable and valid assessment of interference of pain on level of functioning and has been used in a variety of post-mastectomy pain trials [58–60] and in patients with breast cancer pain [61–64]. Given the well-established inverse relationship between pain and quality of life [65, 66], we will use the EQ-5D-5L [67], which is a widely used measurement tool for assessing health-related quality of life [68]. This instrument is also commonly used in health economic evaluations, which will be explored if lidocaine infusions are shown to be significant for the primary outcome.

Cancer recurrence will be assessed at 3 and 12 months. If cancer recurrence is suspected during follow-up, RAs will review with their respective Site Principal Investigator (PI) and send relevant source documents to the coordinating center. If needed, this outcome will be sent for adjudication by two independent investigators blinded to the patient’s treatment allocation.

Adverse events (AE) will be monitored as a secondary safety outcome. Adverse events will be defined as “any noxious and unintended response to an Investigation Medicinal Product (IMP) related to any dose.” Given the half-life of lidocaine is approximately 90 min, screening for adverse events will be limited to the period of administration (intraoperative period), and immediate postoperative period (up to 24 h after surgery). AEs will be screened and reported by the attending anesthesiologists administering study medications intraoperatively and by RAs reviewing postoperative medical records. Serious adverse events (SAEs) will be monitored throughout the entire trial conduct.

Cost-effectiveness will be assessed if lidocaine significantly reduces persistent pain at 3 months to determine cost-savings from a patient and societal perspective if lidocaine is adopted widely into practice.

### Recruitment and participant timeline

We will use recruitment strategies that we developed in our pilot trial and prior perioperative studies [36, 69]. We aim to approach patients through involved breast surgeon offices, as well as screening daily preoperative assessment clinic lists, operating room booking lists, incoming patient lists on surgical wards, and monitor operating room procedure lists for add-on surgeries. Study RAs will approach eligible patients to verify eligibility, either in person or remotely, and to obtain voluntary informed consent. In some cases, where it may not be possible for the prospective participant to sign the informed consent form, verbal consent will be obtained and baseline questionnaires will be provided to the patient. The expectation remains that the consent form will be signed and documented in-person prior to surgery. Patients may also sign the consent form online via REDCap and no study interventions will occur prior to written consent. Further, patients have the right to withdraw from the study at any time for any reason, without the need to justify their decision.

RAs will randomize patients through an online 24-h, interactive, web-based randomization system. Randomization will occur on the day of surgery to minimize the risk of surgery being postponed or cancelled after randomization. For logistical reasons, sites have the ability to randomize the day before to allow ample time for study drug preparation, or on a Friday for a Monday case.

Study patients will be followed up in the post-anesthesia care unit (PACU) within 1 h of surgery, on PODs 1 to 3, and at 3 and 12 months after surgery. Sites can follow-up with patients electronically via approved online platforms (e.g., MS Teams, Zoom), traditional data collection methods (i.e., telephone follow-up), or both. REDCap allows electronic follow-up surveys which can be used to collect self-reported data. A secure patient-specific link is sent via email or text message to collect self-reported data on PODs 1, 2, and 3 (pain scores, opioid consumption, and adverse events). Patients that follow-up via traditional methods will be trained to complete a paper pain diary before hospital discharge to record their acute pain (pain at rest and movement) scores, opioid consumption, and any adverse events. RAs will call patients on POD 1 to remind them to complete their pain diary and again on POD 4 to obtain data from their pain diary over the phone—this method was used in the pilot and achieved > 98% data collection [36]. For data corroboration, we will ask patients to bring their paper pain diary to their next visit with their surgeon or to submit it electronically. The 3- and 12-month follow-up visit will occur electronically or via a telephone interview [70]. To ensure retention at the 3- and 12-month follow-ups, we obtain contact information from patients, including their relatives and care givers. If we do not hear from a patient at the 3- or 12-month follow-up, we will mail the questionnaires to their home with a pre-paid envelope to complete and return to the site recruitment center.

### Unblinding

In cases of suspected lidocaine toxicity, patients will be treated according to standard clinical algorithms for the management of lidocaine toxicity as described by the American Society of Regional Anesthesia [71, 72]. This algorithm includes the administration of intralipid, the antidote for lidocaine toxicity. Intralipid is a lipid emulsion with a high safety profile available at all involved clinical sites in this trial. Intralipid has no known adverse reactions in those without lidocaine overdose, and as such, intralipid will be administered only on suspicion of lidocaine toxicity without unblinding patient’s group allocation. If circumstances arise requiring unblinding of patient’s group allocation, local site investigators will contact the Coordinating Centre to discuss unblinding decisions jointly. All efforts will be made to avoid unnecessary unblinding and the decision to unblind will be made on a case-by-case basis.

### Sample size

Only a few prospective studies provide data on the prevalence of persistent pain at 3 months after breast cancer surgery, with estimates ranging from 33 to 82% [32, 34, 49, 73, 74]. The largest study (*n* = 150) reported a 33% prevalence at 3 months; however, only 84 (56%) patients provided follow-up data. Our pilot (*n* = 100), conducted at two Canadian centers, found a prevalence of persistent pain of 58.3% (95% CI 38.6 to 78.1%) in the control group at 3 months. Furthermore, our pilot found a 32% relative risk reduction (RRR) with the use of IV lidocaine, whereas Grigoras et al. [32] and Kim et al. [34] found a 75 and a 50% RRR at 3 months, respectively. We believe these RRR estimates are likely overestimations of the true treatment effect given the small size of the trials, the multiple pathogenic mechanisms involved, and the rarity of such large effect sizes in the medical literature [75, 76] and within chronic pain prevention trials [77].

We have therefore chosen to design our trial to identify a plausible, but clinically important, 25% RRR while using 30% as the control group pain prevalence at 3 months. To achieve 90% power to detect in a two-sided comparison with *α* = 0.05, and 10% loss-to follow-up, we will need a sample of 1602 participants. For a fixed RRR, power increases as the control group prevalence increases. This sample size also provides adequate power to inform realistic RRRs in important but less common secondary outcomes such as presence of moderate-to-severe pain. For example, based on a control group prevalence of moderate-to-severe pain of 16% in our pilot, we have 80% power to detect a RRR of 31% or greater.

### Analysis

A statistical analysis plan (SAP) has not yet been completed and approved by the Steering Committee. In general, the analysis of results will be completed in the following manner but is subject to minor changes in the SAP. Patients will be analyzed according to their allocated treatment group (intention-to-treat analysis). No imputations will be made for missing data. The relative risk for the primary outcome of the presence of persistent pain at 3 months will be estimated (along with its 95% CI) from a log-binomial model that includes factors for treatment group and the stratification variables (study center, breast reconstruction). If the number of centers is large, a center will be represented by a random effect [78]. A similar analytical approach will be used for secondary binary outcomes. Treatment effects will also be quantified by calculating absolute differences between intervention groups. Analyses of continuous variables such as acute pain NRS scores at rest and movement, oral morphine-equivalent opioid consumption, time to hospital discharge, scores on SF-MPQ-2, POMS, BPI, and EQ-5D-5L will use linear regression (with transformations of outcomes if necessary) to estimate treatment effects and their 95% CIs, again with factors (or random effects) for stratification variables. Baseline characteristics in each group will be summarized without any comparative analyses. If more than 5% of participants are missing the primary outcome, the primary analysis will combine results across multiple imputed datasets, generated from a regression model using other available pain measurements. Analysis of adverse events will be summarized using count and proportions in each intervention group (lidocaine and placebo groups) and numerical values will be used for exploratory analysis between groups. All statistical analyses will be performed using the R statistical language.

Analysis of trial outcomes will be completed at the end of the trial. There will be no interim analysis for efficacy due to the possibility of introducing bias and over-estimating treatment effects [79–81].

We plan to conduct three a priori subgroup analyses to determine whether there is a differential effect of an intraoperative lidocaine infusion on (1) those who underwent a mastectomy versus a lumpectomy; (2) those who underwent an axillary lymph node dissection versus a sentinel lymph node biopsy/no lymph node removal; (3) those who received radiotherapy versus those who did not. The corresponding analyses will use the log-binomial model and include an interaction between the subgroup and treatment variables. We hypothesize that patients with mastectomies and axillary lymph node dissections will benefit more than their respective comparators given that these procedures are associated with greater surgical-induced nerve injury and, thus, may be more responsive to the effect of lidocaine [10]. We also expect that patients not receiving radiotherapy would demonstrate a greater effect of lidocaine since those who receive radiotherapy receive their treatment postoperatively, exposing them to a source of nerve injury that cannot be mitigated through the use of lidocaine during surgical resection.

We will conduct a within-trial cost-effectiveness analysis, using societal and patient perspectives and the trial time horizon (12 months). For general health-related quality of life, we will use the EQ-5D-5L [82, 83] and for healthcare costs we will use a structured healthcare use survey. Unit costs for healthcare services will be obtained from the respective Canadian sources [84, 85]. We will follow the Canadian economic evaluation guideline [86]. The cost-effectiveness will be estimated by the incremental cost-effectiveness ratio (ICER) measuring the cost per additional quality-adjusted life year (QALY) when comparing the lidocaine arms to placebo. We will also conduct incremental net-benefit (INB) analysis using accepted willingness-to-pay thresholds. Uncertainty in cost-effectiveness analysis will be evaluated by bootstrapping and by cost-effectiveness acceptability curves [87]. Analysis will be conducted only if lidocaine is found to significantly reduce persistent pain at 3 months (primary outcome).

## Discussion

### Data management

Trial data will be collected and stored on the secure, web-based REDCap system. This data collection platform has been widely used for research within academia and industry around the world. To ensure data quality, training sessions will be provided to all research personnel involved in the trial. Standardized CRFs will be created in REDCap for collecting baseline outcome data, with real-time checks for validity and completeness.

All data will be centrally monitored for this study, and the central monitoring process will be in conjunction with procedures such as investigator’s training and meetings, and extensive written guidance to assure the conduct of the trial is in accordance with Good Clinical Practice (GCP) Guidelines. During the central monitoring process, the monitor will assess study files and documentation against GCP standards, regulatory requirements (e.g., investigator qualifications and documented training), standard operating procedures (SOPs), and any study-specific SOPs. The monitor will also assess protocol adherence (e.g., verify source documents), recruitment (e.g., verify informed consent), drug accountability (e.g., storage, use, return). All observations noted during the monitoring review will appear in the monitoring report and associated follow-up site communication. In the event that an issue is identified, through data or other means, then a teleconference to discuss the issues will be scheduled. If the issue cannot be resolved with the site personnel, the issue will be escalated to the PI. If the issue is not resolved or requires further investigation, then an on-site monitoring visit may be conducted.

### Data access and confidentiality

User privileges in the REDCap system will require active usage; otherwise, access automatically relapses after 6 months of inactivity. REDCap also implements authentication to validate the identity of end-users that log into the system. After completion of recruitment and all follow-ups, trial data will be stored on a secured online cloud-based repository for analysis on the primary and secondary outcomes. All trial data will be available to trial PIs and ACTU. The trial dataset can be made available upon request by individual recruitment sites through a data sharing agreement for proposed secondary analyses on the dataset. All secondary analyses will have to be reviewed and approved by the Steering Committee. Active maintenance of the data repository will be governed by the ACTU and Sponsor. All trial data will be de-identified and anonymized using REDCap, which generates a study identifier that cannot be linked to the participant’s name or personal information. This is done to protect identities of enrolled participants, in keeping with the Personal Health Information Protection Act.

### Independent data safety and monitoring board

An Independent Data Safety and Monitoring Board (IDSMB) has been established, composed of a methodologist, pain physician and researcher, and statistician. The IDSMB will meet after every 400 patients are recruited and at the end of the trial. They will review the unblinded safety and efficacy (if requested) data provided to them by the IDSMB-Reporting Statistician to make recommendations to the Principal Investigators and Steering Committee. The IDSMB will provide recommendations about stopping the study based on considerations of harm associated with the treatment. There are no specific stopping boundaries used but at an interim safety analysis, the IDSMB members will review serious adverse events, both expected and unexpected, to determine if there is a need to stop the trial early due to harm. In addition, the IDSMB may make observations or recommendations to the sponsor about, but not limited to, the following: definitions of and responses to adverse events and patterns in adverse events; benefit/risk ratio of procedures and participant burden; selection, recruitment, and retention of participants; adherence to protocol requirements; completeness, quality, and analysis of measurements; amendments to the study protocol and consent forms; performance of individual centers and core labs; and participant safety.

### Patient engagement

A Patient Advisory Committee (PAC) comprised of representatives from patient organizations and patients with lived experience of chronic pain will meet quarterly to discuss and provide input in the trial conduct, analysis, knowledge translation, and mobilization efforts. A member of the PAC will also sit on the trial Steering Committee to ensure that patient views, concerns, needs, and preferences are incorporated and part of all major decisions pertaining to the trial.

### Ethics and regulatory approval

We are currently recruiting at 14 clinical centers across five different provinces in Canada. We have obtained ethics approval for these sites (date of approval): Mount Sinai Hospital in Toronto, Canada (May 7, 2021); University Health Network in Toronto, Canada (April 7, 2021); Women’s College Hospital in Toronto, Canada (June 25, 2021); St. Michael’s Hospital in Toronto, Canada (September 1, 2021); Juravinski Hospital in Hamilton, Canada (June 2, 2021); Sunnybrook Health Sciences Centre in Toronto, Canada (June 11, 2021); Humber River Hospital in North York, Canada (Dec 3, 2021); The Ottawa Hospital in Ottawa, Canada (October 5, 2021); Maisonneuve-Rosemont Hospital in Montreal, Canada (July 30, 2021); Foothills Medical Centre in Calgary, Canada (November 16, 2021); Health Sciences Centre - Eastern Health in St. John’s Canada (September 8, 2023); IWK Health Centre in Halifax, Canada (April 26, 2023); Thunder Bay Regional Health Sciences Centre in Thunder Bay, Canada (February 26, 2024); and Sturgeon Community Hospital in St. Alberta, Canada (July 28, 2023).

At the time of writing, we are also in the process of activating additional sites including international sites. We will be obtaining ethics approval prior to the start of recruitment at these sites.

Given that intravenous administration is an off-label route on the product monograph of lidocaine, we needed to obtain Health Canada approval prior to conducting this trial. Any harm to patients occurred during the trial will be covered by the local institution. We obtained approval from Health Canada for all our Canadian recruitment sites on February 4, 2021 (NOL ID HC6-24-c248198). We will obtain approval from relevant national regulatory agencies for the international sites that may participate. Any additional protocol amendments will be reviewed and approved by local research ethics boards and Health Canada before implementing changes into the clinical trial.

### Knowledge translation

Integrated Knowledge Translation (IKT) strategies will include empowering knowledge users on our investigative team (i.e., anesthesiologists, breast surgeons, pain medicine clinicians) to include trial results into their practice and disseminate to their respective institutions. As part of IKT and end-of-grant KT strategies, we will disseminate our findings to practitioners, patients, the public, and other knowledge users via publishing the results in peer-reviewed medical journals, a coordinated social media strategy involving influential online stakeholder groups, media relations with professional anesthesia and surgery associations (e.g., Canadian Anesthesiologists Society, American Society of Anesthesiologists, Canadian Breast Cancer Network, American Society of Breast Surgeons), and presentations at national and international conferences. We will also advocate for inclusion of trial results in updated clinical practice guidelines. All co-investigators and collaborators involved during the recruitment of trial patients, those involved in the design and development of trial protocol, and those involved in the analysis of results will be included as co-authors in the publication of the main trial results. There are currently no plans for making full trial results publicly available for open access.

### Consent for publication

Not applicable —no identifying images or other personal or clinical details of participants are presented here or will be presented in reports of the trial results. The participant information materials and informed consent form are attached as supplementary materials.

## Trial Status

The current operational protocol version number is 3.0 that was approved on March 25, 2024. Recruitment began on September 22, 2021. The approximate date when recruitment is expected to be completed in mid-2025.

### Supplementary Information

Supplementary Material 1.

Supplementary Material 2.

## Abbreviations

ACTU: Anesthesia Clinical Trials Unit
AE: Adverse events
BPI: Brief Pain Inventory
CI: Confidence Interval
CRFs: Case Report Forms
DN4: Douleur Neuropathique 4
DSMB: Data Safety and Monitoring Board
EQ-5D-5L: EuroQol- 5 Dimension- 5 Level
GCP: Good Clinical Practice
ICER: Incremental cost-effectiveness ratio
IASP: International Association for the Study of Pain
IDSMB: Independent Data Safety and Monitoring Board
IKT: Integrated Knowledge Translation
IMMPACT: Initiative on Methods, Measurement, and Pain Assessment in Clinical Trials
IMP: Investigative Medicinal Product
INB: Incremental net-benefit
IV: Intravenous
KT: Knowledge translation
NRS: Numeric Rating Scale
OR: Operating room
PACU: Post-anesthesia care unit
PCS: Pain Catastrophizing Scale
PI: Principal Investigator
PMPS: Post-mastectomy pain syndrome
POD: Postoperative day
POMS: Profile of Mood States
QALY: Quality-adjusted life year
RA: Research assistant
RCT: Randomized controlled trial
RR: Relative risk
RRR: Relative risk reduction
SAE: Serious adverse event
SAP: Statistical analysis plan
SF-MPQ-2: Short Form McGill Pain Questionnaire 2
SGBA +: Sex- and Gender-Based Analysis Plus
