# Supplementary material for: Prevention of persistent pain with lidocaine infusions in breast cancer surgery (PLAN): study protocol for a multicenter randomized controlled trial
Source: Trials. 2024 May 22;25:337. doi: 10.1186/s13063-024-08151-4 (PMC11110187; doi:10.1186/s13063-024-08151-4)
Supplement: Supplementary file 1 — Supplementary Material 1. [file 13063_2024_8151_MOESM1_ESM.docx]

**Appendix**

Appendix A: Informed Consent Form


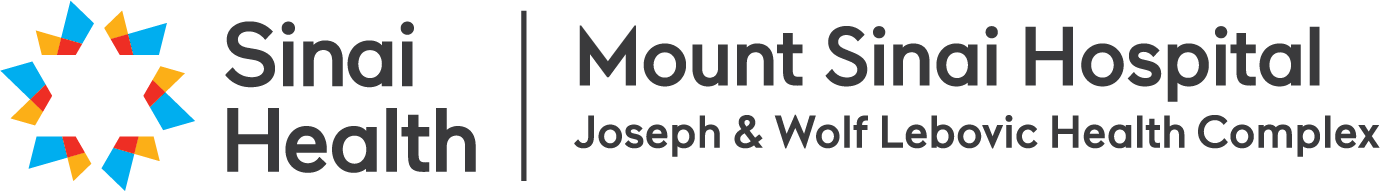


Study Information and Informed Consent Form

Study Title: **P**revention of persistent pain with **L**idoc**A**ine i**N**fusions in breast cancer surgery (**PLAN**) Trial

Official Study Title for Internet Search on <http://www.ClinicalTrials.gov>: Prevention of persistent pain with lidocaine infusions in breast cancer surgery

Trial Code/study #: **PLAN Study**

**Study Doctor:** Dr. James Khan

**Sponsor:** Dr. James Khan, MD

**EMERGENCY contact number** (24 hours/day 7 days/week): 647-781-9540

Non-Emergency contact numbers are at the end of this document in the “Where can I get more information?” section.

Overview and Key Information

1. What am I being asked to do?

We are inviting you to take part in a research study. We do research studies to try to answer questions about how to prevent, diagnose, and treat diseases or side effects from treatment of diseases like cancer.

We are asking you to take part in this research study because you have breast cancer and will undergo breast cancer surgery as part of your treatment.

1. Taking part in this study is your choice.

You can choose to take part or you can choose not to take part in this study. You also can change your mind at any time. Whatever choice you make, you will not lose access to your medical care or give up any legal rights or benefits.

This document has important information to help you make your choice. Take time to read it. Talk to your doctor, family, or friends about the risks and benefits of taking part in the study. It is important that you have as much information as you need and that all your questions are answered. See the “Where can I get more information” section.

1. Why is this study being done?

Surgery is a critical part of breast cancer treatment. A common complication after breast cancer surgery is persistent pain that does not go away after the expected time needed to heal from a surgical procedure (e.g. 3 months). Unfortunately, about 40% of patients who have breast cancer surgery will develop persistent pain and approximately 18% will suffer from severe pain. This is due to the many nerves within the breast tissue that can be unintentionally cut or damaged during surgery. Chemotherapy and radiotherapy after surgery can further injure these nerves. Nerve injury can lead to the development of long-term chronic pain that can persist for several years after surgery.

While there are no known interventions that prevent persistent pain after breast cancer surgery, there is published research suggesting that an infusion of lidocaine during surgery can prevent the development of persistent pain. Lidocaine is commonly used to prevent acute pain after surgery.

This study is being done to answer the following question: Can giving lidocaine as an infusion during surgery to breast cancer patients reduce the amount of pain a patient has when recovering from their surgery and improve quality of life?
We are doing this study because we want to find out if this approach is better or worse than the current standard of care for managing pain after surgery for your breast cancer. Standard of care is defined as care most people get for managing pain after breast cancer surgery.

1. What is the standard of care for managing my pain from surgery?

Currently, there are no specific pain management therapies that are given to prevent persistent pain after surgery. Certain pain medications and therapies (i.e., nerve blocks) are aimed to alleviate acute pain (pain immediately after surgery). Lidocaine is administered at the discretion of the anesthesiologist during surgery, but there is no current standard practice.

1. What are my choices if I decide not to take part in this study?

You do not have to take part in this study. If you decide not to participate in this study, you will proceed with your preoperative clinic visit, surgery, and follow-up visits as per the hospital’s standard of care practices.

1. What will happen if I decide to take part in this study?

If you decide to take part in this study, you will either get lidocaine or placebo during the surgery. After you finish your study treatment, the study research staff will continue to follow you and watch you for side effects. This will be done 1 hour and 1-3 days after surgery. Follow up will continue for a year, at 3 months and 1 year after surgery.

Even if your participation in the study ends earlier than required, the study team will still collect information from your chart and continue to follow you for the duration of the study.

1. What are the risks and benefits of taking part in this study?

There are both risks and benefits to taking part in this study. It is important for you to think carefully about these as you make your decision.

Risks

We want to make sure you know about a few key risks right now. You can find more information in section 13: “What risks can I expect from taking part in this study?”

There is a risk that you could have side effects from the study lidocaine infusion during surgery. These side effects may or may not be different from what you would get with the standard of care.

Lidocaine infusions in the dosages and duration used in this study are rarely associated with any adverse reactions. Some of the most common side effects that the study doctors know about are:

- Nausea/Vomiting
- Dizziness
- Low blood pressure
- High blood pressure
- Low heart rate

There may be some risks that the study doctors do not yet know about.

Benefits

There is no guarantee that you will receive any benefit from taking part in the study. This study may help the study doctors learn things that may help other people in the future.

1. If I decide to take part in this study, can I stop later?

You can decide to stop taking part in the study at any time. If you decide to stop, let your study doctor know as soon as possible. If you stop, you can decide if you would like the study doctor to continue to contact you to find out how you are doing.

You may withdraw your permission to use your personal health information for this study at any time by letting the study doctor know. However, this would also mean that you withdraw from the study. Your study data that was recorded before you withdrew will be used but no information will be collected or sent to the sponsor after you withdraw your permission.

Your study doctor will tell you in a timely manner about new information or changes in the study that may affect your health or your willingness to continue in the study.

1. Are there other reasons why I might stop being in the study?

The study doctor may stop your participation in the study early, if:

- Your health changes and the study is no longer in your best interest or you are unable to tolerate the study treatment.
- New information becomes available and the study is no longer in your best interest.
- The study is stopped by Health Canada, the Research Ethics Board (REB), or study sponsor. The study sponsor is the person or organization who oversees the study.

---------------------------------------- End of Overview Section ----------------------------------

**It is important that you understand the information in the Informed Consent Form before making your decision.** Please read, or have someone read to you, the rest of this document. If there is anything you don’t understand, be sure to ask your study doctor or nurse.

1. What is the purpose of this study?

The purpose of this study is to test if lidocaine infusion during surgery can reduce the amount of pain a patient has when recovering from their surgery and improve quality of life. The effects of lidocaine will be compared to a placebo. A placebo is an infusion that looks like the study drug, but contains no medication. There will be about 1200 people taking part in this study.

1. What are the study groups?

This study has 2 study groups. You will not be told which group you are in.

We will use a computer to assign you to one of the study groups. This process is called “randomization.” It means that your doctor will not choose and you cannot choose which study group you are in. You will be put into a group by chance. However, this information will be available in case of an emergency.

You will have an equal chance of being in the **Intervention Group** or in the **Control Group**

- **Intervention group**: Patients will receive lidocaine through a vein in the arm (IV).
- **Control group:** Patients will receive a placebo (normal saline solution) through a vein in the arm (IV). A placebo liquid looks like the study drug, but contains no medication.

In the operating room, the anesthesiologist will give patients an initial single injection (bolus) dose of lidocaine or placebo at the beginning of surgery, followed by a steady infusion of lidocaine or placebo throughout the procedure, up to 30 minutes after the end of surgery.

All other pain management plans during and after surgery will be left to the discretion of your physicians.

Another way to find out what will happen to you during this study is to read the chart below. Start reading at the left side and read across to the right, following the lines and arrows:

Randomize –

The computer will randomly put you in a study group.

**Control Group**

Placebo injection + infusion until the end of surgery and up to 30 minutes in recovery

You agree to take part in the study and sign this consent form.

**Intervention Group**

Lidocaine injection + infusion until the end of surgery and up to 30 minutes in recovery

1. **What exams, tests, and procedures are involved** **in this study?**

Listed below are exams, tests, and procedures that need to be done as part of this study to monitor your safety and health, but may not be included in the standard of care. We will use them to carefully follow the effects of the study treatment, including preventing and managing side effects.

The Questionnaires and diaries described below will be provided in paper or electronically through REDCap which is a secure online system situated at University Health Network (UHN).  This online system will be used to enter other data that has been collected for study purposes.

**Questionnaires**

If you choose to take part in this study, you will be asked to fill out forms with questions about pain intensity, your physical and emotional well-being and quality of life and mood. Researchers will use this information to learn more about how pain affects people.

Since these forms are being used for research, the responses you provide will not be shared with your study doctor. You do not have to answer any question that makes you feel uncomfortable. If you have any serious health issues or other concerns, please talk with your doctor or nurse right away.

You will be asked to fill out the study questionnaire forms at the following times:

- Before surgery:

1. A questionnaire to rate your present pain on a scale from 0-10 (at rest and at movement). This can be completed in 1- 5 minutes
2. A questionnaire to understand the type of thoughts and feelings that you have when you are in pain. This can be completed in 5- 10 minutes

- After surgery:

1. At 1 hour after surgery: While in the recovery room, we will ask you to rate your present pain on a scale from 0-10 (at rest and at movement).
2. Day 1, Day 2 and Day 3: We will ask you to rate your present pain on a scale from 0-10 (at rest and at movement) twice a day. Each can be completed in 1- 5 minutes
3. At 3- month and 12-month: Questionnaires will be sent to assess your pain, your physical and emotional well-being, quality of life and mood feelings. These questionnaires will take 15-20 minutes to complete.

Study questionnaires can be completed over the phone, using paper format, or electronically. If you agree, you will receive an email with the link to the online questionnaires. Reminders to complete the questionnaires may also be sent to you by email, by text or through the electronic system.

**Pain Diary**

You will be given a form to list all the pain medication that you are taking after your surgery (Day 1 to Day 3 after surgery). This diary is also available online. You will receive reminders to complete it online via link sent either by email or text message.

**Follow up**

In addition to the completion of study questionnaires, research staff will contact you by phone or email to collect information about your recovery, daily for the first 3 days post-surgery, then at 3-months and 12-months after surgery. We will ask about your current medications, any additional treatment you have received and complications you have experienced, if any.

We may also contact your family physician to collect this information.

**Data Collection**

The data collected for this study will be stored in the Research Electronic Data Capture (REDCap) system which is a web-based application housed at the University Health Network on a secure server. This system was developed by Vanderbilt University to capture data for clinical research and meets all Canadian privacy laws. Data stored in the system will not include any direct participant identifiers.

**Study calendar:**

See the participant study calendar below. It shows how often the study procedures will be done.

|  | **Before surgery** | **During Surgery** | **After Surgery Follow-ups** | | |
| --- | --- | --- | --- | --- | --- |
|  |  |  | **Hour 1** | **Day 1 to 3** | **3 &12 Months** |
| Screen/ Informed Consent/ Eligibility | X |  |  |  |  |
| Receive Study Drug |  | X |  |  |  |
| Questionnaire about pain severity | X |  | X | X | X |
| Questionnaire about feelings and thoughts when in pain | X |  |  |  |  |
| Questionnaire to assess pain caused by nerve damage |  |  |  |  | X |
| Questionnaire to assess pain type |  |  |  |  | X |
| Questionnaire to assess your emotions |  |  |  |  | X |
| Questionnaire to assess physical functioning |  |  |  |  | X |
| Questionnaire to assess your mood and quality of life |  |  |  |  | X |
| Pain Diary | X |  |  | X | X |
| Medical record review | X | X | X | X | X |

1. What risks can I expect from taking part in this study?

The risks and side effects of the standard procedures will be explained to you as part of your standard of care and therefore are not listed.

Drug Risks

The lists below show the most common side effects doctors know about. Keep in mind that there might be other side effects doctors do not yet know about. If important new side effects are found, the study doctor will discuss these with you.

Patient safety is carefully monitored and recorded for any complications of the study interventions. Lidocaine infusions used in the dosages and duration used in this study are rarely associated with any adverse reactions.

**Potential side effects (1% to less than 10%) include:**

- Low or high blood pressure, nausea/vomiting, dizziness, and low heart rate.

**Rare reactions (less than 0.1%) are possible and include:**

- An allergic reaction; diplopia (blurry vision); abnormal heart rhythms; convulsions, and cardiac arrest.

These rare side effects are not expected and are only seen in patients who receive a large dose of lidocaine intravenously, and not with dosages used in this study. An antidote for lidocaine toxicity exists, and if you appear to be having a lidocaine overdose, your clinical care team will provide you with this antidote.

There is also a possibility of risks that we do not know about and have not been recorded in medical literature or seen in study participants to date. You will be very closely monitored during your time at the hospital and any complications will be quickly identified and managed.

1. What are the costs of taking part in this study?

You will not have to pay for the lidocaine or placebo while you take part in this study and the study is ongoing. The costs of your standard of care medical treatment will be paid for by your provincial medical plan to the extent that such coverage is available. You will not be paid for taking part in this study.

1. What happens if I am injured because I took part in this study?

If you are injured as a result of taking part in this study and need medical treatment, please talk with your study doctor right away about your treatment options. In case of research injury or side effects, medical care will be provided or you will be referred for appropriate medical care at no cost to you.

By signing this form you do not give up any of your legal rights against the investigators, sponsor or involved institutions for compensation, nor does this form relieve the investigators, sponsor or involved institutions of their legal and professional responsibilities.

1. Who will see my medical information?

Protecting and maintaining confidentiality of your medical information is of critical importance to us. Information about you will have a code and will not show your name or address, or any information that directly identifies you. All information collected during this study, including your personal health information (PHI) will be kept confidential and will not be shared with anyone outside the study unless required by law. You will not be named in any reports, publications, or presentations that may come from this study.

The study doctor will keep any personal health information (PHI) about you in a secure and confidential location for a minimum of 25 years as required by research standards. A list linking your study number with your name will be kept by the study doctor in a secure place, separate from your study file.

In case any serious adverse events are believed to be related to this research study, personal health information (PHI) will be removed, and the appropriate data will be sent to the study sponsor (Dr. Khan) and relevant regulatory agencies for the purpose of product safety.

There are organizations and their representatives that may look at or receive copies of some of the information in your study records for data analysis and quality assurance. Your health information in the research database also may be shared with these organizations. They must keep your information private, unless required by law to give it to another group. Some of these organizations are:

- The study sponsor (Dr. James Khan) or one of his delegated representatives
- The Ontario Cancer Research Ethics Board (REB), which is a group of people who review the research with the goal of protecting the people who take part in the study.
- Health Canada because they oversee the use of drugs in Canada.

1. Conflict of Interest

This center is receiving funds from a grant from the Government of Canada’s health research investment agency, the Canadian Institutes of Health Research (CIHR) to help offset the costs of conducting this research.

The doctor treating you also may be the doctor in charge of this study.

If you would like additional information about the funding for this study, or about the role of the doctor in charge of this study, please speak to the study staff or to the ethics board.

1. Where can I get more information?

A description of this clinical trial will be available on [*http://www.ClinicalTrials.gov*](http://www.ClinicalTrials.gov)*.* This website will not include information that can identify you. You can search this website at any time.

If you have questions about taking part in this study, or if you suffer a research-related injury, you can talk to your study doctor, or the doctor who is in charge of the study at this institution. That person is:

Dr. James Khan 416-586-5270 (james.khan@medportal.ca)

For questions about your rights while in this study, call the: Office of the Chair of the Ontario Cancer Research Ethics Board at: 416-673-6648 OR Toll free: 1-866-678-6427 ext. 6648

**My signature agreeing to take part in the study**

********NOTE: You may also be able to review and sign this Consent form online. The study staff will provide you with a link to do this, if this option is available to you.

I have read this consent form or had it read to me. I have discussed it with the study doctor/delegated research staff and my questions have been answered. I will be given a signed and dated copy of this form. I agree to take part in the study.

_________________________________ ____________________________ ___________________

Signature of Participant Printed Name Date

_________________________________ ____________________________ ___________________

Signature of Person Conducting Printed Name Date

the Consent Discussion

Participant Assistance

**Complete the following declaration only if the participant is unable to read:**

- The informed consent form was accurately explained to, and apparently understood by, the participant, and,
- Informed consent was freely given by the participant.

____________________________ ________________________ _________________

Signature of Impartial Witness Printed Name Date

**Complete the following declaration only if the participant has limited proficiency in the language in which the consent form is written and interpretation was provided as follows:**

- The informed consent discussion was interpreted by an interpreter, and,
- A sight translation of this document was provided by the interpreter as directed by the research staff conducting the consent.

Interpreter declaration and signature: By signing the consent form I attest that I provided a faithful interpretation for the discussion that took place in my presence, and provided a sight translation of this document as directed by the research staff conducting the consent.

____________________________ ________________________ _________________

Signature of Interpreter Printed Name Date
